# Limited diagnostic performance of imaging evaluation for staging in gastric-type endocervical adenocarcinoma: a multi-center study

**DOI:** 10.1007/s11604-024-01713-1

**Published:** 2024-12-03

**Authors:** Yuki Himoto, Aki Kido, Koji Yamanoi, Yasuhisa Kurata, Satoshi Morita, Nao Kikkawa, Hideyuki Fukui, Ayumi Ohya, Yuko Iraha, Takahiro Tsuboyama, Kimiteru Ito, Yasunari Fujinaga, Sachiko Minamiguchi, Masaki Mandai, Yuji Nakamoto

**Affiliations:** 1 Department of Diagnostic Imaging and Nuclear Medicine, Kyoto University Graduate School of Medicine, Shogoinkawahara-Cho 54, Sakyo-Ku, Kyoto, 606-8507 Japan; 2 Department of Gynecology and Obstetrics, Kyoto University Graduate School of Medicine, Kyoto, Japan; 3 Department of Biomedical Statistics and Bioinformatics, Kyoto University Graduate School of Medicine, Kyoto, Japan; 4 Department of Diagnostic Radiology, National Cancer Center Hospital, Tokyo, Japan; 5 Department of Diagnostic and Interventional Radiology, Osaka University Graduate School of Medicine, Osaka, Japan; 6 Department of Radiology, Shinshu University School of Medicine, Nagano, Japan; 7 Department of Radiology, Graduate School of Medical Science, University of the Ryukyus, Okinawa, Japan; 8 Department of Diagnostic Pathology, Kyoto University Graduate School of Medicine, Kyoto, Japan

**Keywords:** Gastric-type endocervical adenocarcinoma, Diagnostic performance, Preoperative imaging, Multi-center study, Staging

## Abstract

**Purpose:**

The purposes of the study are to assess the diagnostic performance of preoperative imaging for staging factors in gastric-type endocervical adenocarcinoma (GEA) and to compare the performance for GEA with that of usual-type endocervical adenocarcinoma (UEA) among patients preoperatively deemed locally early stage (DLES) (< T2b without distant metastasis).

**Materials and methods:**

For this multi-center retrospective study, 58 patients were enrolled. All had undergone MRI with or without CT and FDG PET-CT preoperatively and had been pathologically diagnosed with GEA at five institutions. Based on the medical charts and radiological reports, the diagnostic performances of preoperative imaging for the International Federation of Gynecology and Obstetrics staging factors were assessed retrospectively. Next, the imaging performance was assessed in preoperatively DLES-GEA (*n* = 36) and DLES-UEA (*n* = 136, with the same inclusion criteria). The proportions of underestimation of GEA and UEA were compared using Fisher’s exact test.

**Results:**

Imaging diagnostic performance for GEA was limited, especially for sensitivity: parametrial invasion, 0.49; vaginal invasion, 0.54; pelvic lymph node metastasis (PELNM), 0.48; para-aortic lymph node metastasis, 0.00; and peritoneal dissemination, 0.25. Among preoperatively DLES patients, the proportions of underestimation were significantly higher in GEA than in UEA; parametrial invasion, 35% vs. 5% (*p* < 0.01); vaginal invasion, 28% vs. 6% (*p* < 0.01); PELNM, 24% vs. 6% (*p* < 0.05); peritoneal dissemination, 6% vs. 0% (*p* < 0.05).

**Conclusion:**

At present, preoperative imaging diagnostic performance for staging factors in GEA does not meet clinical expectations, especially for sensitivity. Among patients preoperatively DLES, the proportions of underestimation in GEA were significantly higher than in UEA. Future incorporation of approaches specifically emphasizing GEA is desirable to improve imaging performance.

## Introduction

Adenocarcinoma, human papillomavirus (HPV)-independent, gastric type, of the uterine cervix (gastric-type endocervical adenocarcinoma, GEA) is a rare histologic subtype of uterine cervical adenocarcinoma, newly included in the 2014 World Health Organization classification, accounting for 10–15% of uterine cervical adenocarcinomas worldwide and 20–25% in Japan [1, 2]. It is not related to HPV infection [1]. In fact, it exhibits gastric-type differentiation [2]. In contrast to usual-type adenocarcinoma (UEA, one of the subtypes of HPV-associated adenocarcinoma), GEA has very aggressive biologic nature, exhibiting extrauterine spread, and a poorer prognosis [3, 4]. Although no standard treatment for GEA has been established yet, GEA has been reported as resistant to chemotherapy and radiation [3, 5]. Therefore, complete surgical resection based on the accurate preoperative evaluation of its extent might be crucially important as a treatment strategy of GEA [6].

Regarding the pretreatment evaluation of cervical cancer, in addition to ultrasound, tomographic imaging modalities play important roles: magnetic resonance imaging (MRI), computed tomography (CT), and fluorodeoxyglucose positron emission tomography (FDG PET)-CT [7]. Among them, MRI has been regarded as the best modality for assessing local invasion into the parametrium, vagina, bladder/rectum, adnexa, and peritoneum in the pelvis. Particularly, CT and FDG PET-CT are used clinically for evaluating pelvic/paraaortic lymph node metastasis (PELNM/PALNM), distant metastases, and peritoneal dissemination. Especially, FDG PET-CT has been regarded as having the highest accuracy for lymph node metastasis (LNM). The International Federation of Gynecology and Obstetrics (FIGO) 2018 guideline, recently updated, has approved the incorporation of imaging evaluation for staging [8]. The role of imaging has become increasingly prominent, with treatment decisions often made in reference to pretreatment imaging.

Despite the established utility of imaging for the pretreatment evaluation of cervical cancer, among patients with GEA, the difficulties of accurate imaging evaluation of tumor extent are often experienced in clinical settings, reportedly because of its infiltrating growth pattern [9–11]. Overestimation of the diagnostic performance of imaging can engender inappropriate treatment planning. Because of GEA rarity among cases, the diagnostic performance of imaging for staging factors has not been explored yet.

This multi-center retrospective study evaluated the diagnostic performance of preoperative imaging for staging factors in GEA in an actual clinical setting. In addition, specifically for patients with preoperatively deemed locally early stage (DLES) cancer (T factors lower than T2b), we also conducted comparisons of the imaging diagnostic performances achieved for GEA and UEA, particularly addressing the proportion of underestimation for staging factors.

## Materials and methods

The institutional review board of Kyoto University Hospital provided a unified approval of this multi-center retrospective study, compliant with the Health Insurance Portability and Accountability Act, for both Kyoto University Hospital and National Cancer Center Hospital. The provision of samples and information was also approved by the institutional review board of other three university hospitals. Informed consent was waived.

### Patients

**Fig. 1:**
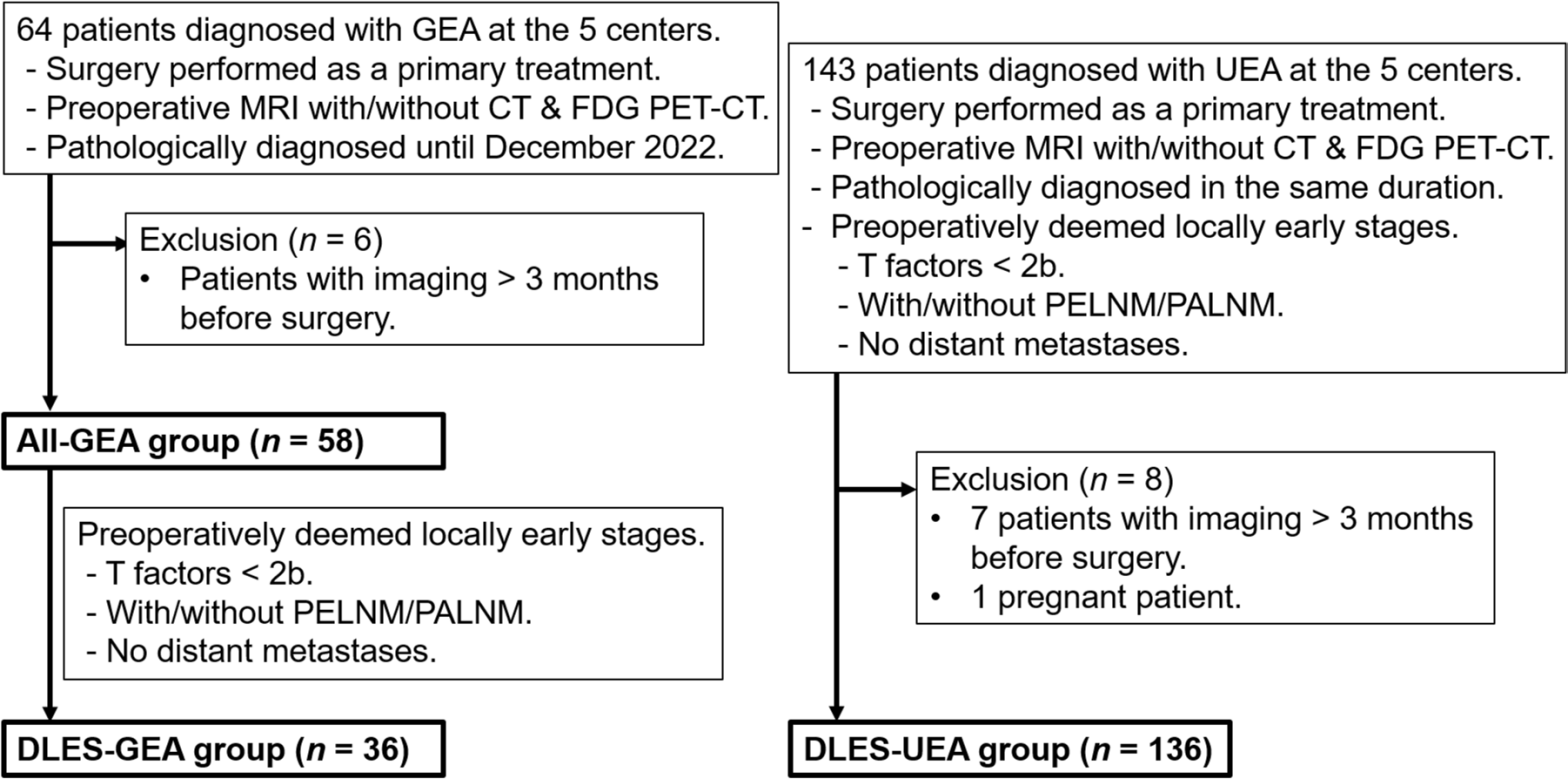
Flowchart of the enrollments of the three groups: All gastric-type endocervical adenocarcinoma (All-GEA) group, deemed locally early stage (DLES)-GEA group, and DLES- usual-type endocervical adenocarcinoma (UEA) group. The start dates differed from center to center, depending on the timing when GEA was introduced in the departments of pathology. PELNM, pelvic lymph node metastasis; PALNM, paraaortic metastasis

Using institutional databases, consecutive patients who met the following inclusion criteria were enrolled. For cases staged according to the FIGO staging system before 2018, reclassification was performed based on FIGO 2018 [8]. Similarly, pathological staging was reclassified following American Joint Committee on Cancer Tumor, Node, Metastasis (AJCC TNM) classification ver. 9 [12]. The inclusion criteria were patients (1) diagnosed with GEA or UEA based on pathological reports from hysterectomies obtained from the electronic medical record systems of each institution by December 2022, and staged as FIGO 2018 IB1 or higher, and (2) who underwent preoperative MRI within three months prior to surgery, regardless of whether CT or FDG PET-CT was performed. Because of the varying timelines during which the pathological diagnosis of GEA was introduced into the departments of pathology, the start dates differ from center to center (2007–2014). After excluding 6 patients with MRI more than three months before the surgery, 58 patients constituted the all-GEA group. Among them, patients preoperatively DLES (clinically lower T factors than 2b) with/without PELNM or PALNM and without distant metastases were categorized as a subgroup of the DLES-GEA group (*n* = 36). For comparison, consecutive patients preoperatively DLES and pathologically diagnosed UEA based on a surgical specimen, and who fulfilled the same inclusion criteria as patients with GEA were sought and included in the DLES-UEA group. After excluding one pregnant patient and 7 patients with MRI more than three months before the surgery, 136 patients constituted the DLES-UEA group (Fig. 1). 15 GEA and 12 UEA patients of Kyoto University Hospital, and all 14 GEA and 18 UEA patients of National Cancer Center Hospital, had been included in the previous studies focusing on the MRI characteristics of GEA, respectively.

### Preoperative imaging evaluations and the pathological confirmations

From medical charts and radiological reports, records of integrated preoperative imaging evaluation of following staging factors were extracted: parametrium, vagina (separately for any invasion and lower third invasion), bladder/rectum (separately for any invasion including muscle invasion without mucosal invasion, and mucosal invasion), PELNM/PALNM, and peritoneum. Although not included in the FIGO staging, adnexal involvement was also added, considering its clinical importance. All were diagnosed by board-certified radiologists expertized in gynecological imaging. When preoperative imaging evaluations of some factors were not clearly described, radiologists of respective centers expertized in gynecologic imaging evaluated them, blinded to information other than the diagnosis of cervical adenocarcinoma.

The records of pathological evaluation of these factors were also noted from pathological reports of surgical specimens. All were diagnosed pathologically by board-certified pathologists.

### Details of imaging modalities

#### MRI

All patients had undergone preoperative MRI in the supine position using 1.5/3.0 T scanners (Philips Electronics N.V., Amsterdam, Holland; GE HealthCare, Chicago, Illinois, USA; Canon Medical Systems Corp., Tochigi, Japan; Siemens Healthcare Diagnostics, Erlangen, Germany). The antispasmodics were administered intramuscularly before scans, if not contraindicated. All images included axial and sagittal T2-weighted images. Additionally, MRI protocols for cervical cancer at all institutions included oblique T2-weighted images, either perpendicular to the cervical canal (oblique axial; four institutions) or parallel to the cervical canal (oblique coronal; one institution). Except for seven cases, all included axial and/or sagittal diffusion-weighted images (DWI). Among the 194 patients, 169 underwent contrast-enhanced MRI (50 GEA and 119 DLES-UEA patients). One GEA and two DLES-UEA patients had undergone FDG PET-MRI (GE HealthCare, Chicago, Illinois, USA) at one center.

#### CT

Preoperative CT had been performed for 157 patients: 50 GEA (30 DLES-GEA) and 107 DLES-UEA patients. Among them, 152 patients had undergone contrast-enhanced studies in the late portal venous phase: 47 GEA (29 DLES-GEA) and 105 DLES-UEA patients. Multidetector CT scanners with 16–320 detector-rows were used (Philips Electronics N.V., Amsterdam, Holland; GE HealthCare, Chicago, Illinois, USA; Canon Medical Systems Corp., Tochigi, Japan; Siemens Healthcare Diagnostics, Erlangen, Germany). All the patients were diagnosed by board-certified radiologists.

#### FDG PET-CT

Preoperative FDG PET-CT had been performed for 86 patients: 24 GEA (14 DLES-GEA) and 62 DLES-UEA patients (Philips Electronics N.V., Amsterdam, Holland; GE HealthCare, Chicago, Illinois, USA; Canon Medical Systems Corp., Tochigi, Japan; Siemens Healthcare Diagnostics, Erlangen, Germany). All the patients were diagnosed by board-certified radiologists, mainly by radiologists expertized in nuclear medicine.

## Statistical analysis

Considering the pathological evaluations of surgical specimens as the gold standard, accuracy, sensitivity, specificity, positive predictive value (PPV), negative predictive value (NPV) of preoperative imaging evaluation was calculated for the following factors in the all-GEA group, the DLES-GEA group, and the DLES-UEA group: parametrium, vagina (separately for any invasion, and lower third invasion), adnexal involvement, bladder and rectum (separately for any invasion, and mucosal invasion), PELNM/PALNM, and peritoneum.

Between the DLES-GEA and DLES-UEA groups, the differences of the proportions of underestimations of preoperative imaging for respective factors were evaluated using Fisher’s exact test. *p* values < 0.05 were inferred as statistically significant. The analyses were performed using MedCalc® ver. 22.013 (MedCalc Software Ltd., Ostend, Belgium). About the underestimation of parametrial invasion, the power was calculated with alfa = 0.05 using PS Power and Sample Size Calculations ver. 3.1.6 (https://biostat.app.vumc.org/wiki/Main/PowerSampleSize).

## Results

### Patients

The patient characteristics of the all-GEA group, the DLES-GEA group, and the DLES-UEA group are presented in Table 1. The details of the number of patients in the all-GEA group, the DLES-GEA group, and the DLES-UEA group from respective institutions were the following: Kyoto University Hospital, 26, 13 and 40; National Cancer Center Hospital, 14, 10, and 18; Osaka University Hospital, 6, 5, and 55; Shinshu University Hospital, 8, 4, and 7; University of the Ryukyus Hospital, 4, 4, and 16. Of the all-GEA group, two patients were preoperatively diagnosed with lobular endocervical glandular hyperplasia, not assumed GEA. Of these two patients, one was diagnosed pathologically as having T2bN0 (pT2b, pN0) without distant metastasis, based on the primary and secondary surgery with pelvic lymph node dissection. The other patient did not undergo additional surgery with pelvic lymph node dissection and was diagnosed pathologically as having T1b1NX (pT1b1, pNX) without distant metastasis. Except for this case, pelvic lymphadenectomy had been performed in all other cases. 50% of the all-GEA group and 33% of the DLES-GEA group had PELNM. Paraaortic lymphadenectomy was performed in 25 patients of the all-GEA group and 13 patients of the DLES-GEA group. Among them, 6 patients of the all-GEA group and 3 of the DLES-GEA group were pathologically diagnosed as having PALNM, all of whom also had PELNM.

**Table 1:** Patient characteristics

	All GEA group (*n* = 58)	DLES GEA group (*n* = 36)	DLES UEA group (*n* = 136)
Age (y), median (IQR)	49 (43–64)	50.5 (41–66)	41 (36–52)
Pathological T stage	*n* (%)	*n* (%)	*n* (%)
1b	20 (34)	18 (50)	116 (85)
2a	5 (9)	4 (11)	12 (9)
2b	29 (50)	14 (39)	8 (6)
3a	2 (3)	0 (0)	0 (0)
3b	0 (0)	0 (0)	0 (0)
4a	2 (3)	0 (0)	0 (0)
Parametrial invasion	33 (57)	14 (39)	8 (6)
Vaginal invasion			
Upper 2/3	26 (45)	13 (36)	10 (7)
Lower 1/3	2 (3)	0 (0)	0 (0)
Adnexal involvement	13 (22)	7 (19)	1 (1)
Bladder invasion			
Any invasion	5 (9)	0 (0)	0 (0)
Mucosal invasion	2 (3)	0 (0)	0 (0)
Rectal invasion			
Any invasion	2 (3)	1 (3)	0 (0)
Mucosal invasion	0 (0)	0 (0)	0 (0)
Lymph node metastasis			
Pelvis	29 (50)	12 (33)	17 (13)
Paraaorta	6 (10)	3 (8)	0 (0)
Peritoneal dissemination	8 (14)	3 (8)	0 (0)

*GEA* gastric-type endocervical adenocarcinoma, *DLES* deemed locally early stage, *UEA* usual-type endocervical adenocarcinoma, *IQR* interquartile range

In the all-GEA group, bladder invasion was observed more often than rectal invasion (12% vs. 3%). One patient had rectal invasion without mucosal invasion in the DLES-GEA group, but no patients had bladder/rectal invasion in the DLES-UEA group. Peritoneal dissemination was not rare among patients diagnosed with GEA: 14% in the all-GEA group, and 8% even in the DLES-GEA group.

## Diagnostic performance of preoperative imaging

The accuracy, sensitivity, and specificity, PPV, and NPV in the all-GEA group, and the DLES-GEA and the DLES-UEA group are presented, respectively, in Tables 2 and 3.

**Table 2:** Preoperative diagnostic performance of imaging in gastric-type endocervical adenocarcinoma (*n* = 58)

Factor	Accuracy	Sensitivity	Specificity	PPV	NPV
Parametrial invasion	38/58, 66% (52–78)	16/33, 49% (31–66)	22/25, 88% (69–97)	16/19, 84% (60–100)	22/39, 56% (40–72)
Vaginal invasion					
Any invasion	44/58, 76% (63–86)	15/28, 54% (34–72)	29/30, 97% (83–99)	15/16, 94% (70–100)	29/42, 69% (53–82)
Lower one third	55/58, 95% (86–99)	0/2, 0% (0–84)	55/56, 98% (90–100)	0/1, 0%	55/57, 96% (96–97)
Adnexal involvement	46/57, 81% (68–90)	2/13, 15% (2–45)	44/44, 100% (16–100)	2/2, 100% (16–100)	44/55, 80% (67–90)
Bladder invasion					
Any invasion	53/58, 91% (81–97)	2/5, 40% (5–85)	51/53, 96% (87–100)	2/4, 50% (15–85)	51/54, 94% (89–97)
Mucosal invasion	57/58, 98% (91–100)	1/2, 50% (1–99)	56/56, 100% (94–100)	1/1, 100% (3–100)	56/57, 98% (91–100)
Rectal invasion					
Any invasion	56/58, 97% (88–100)	0/2, 0% (0–84)	56/56, 100% (94–100)	0/0, NA	56/58, 97% (88–100)
Mucosal invasion	NA	NA	NA	NA	NA
Lymph node metastasis					
Pelvic lymph node	40/57, 70% (57–82)	14/29, 48% (29–67)	26/28, 93% (77–99)	14/16, 88% (62–98)	26/41, 63% (47–78)
Paraaortic lymph node	18/25, 72% (51–88)	0/6, 0% (0–46)	18/19, 95% (74–100)	0/1, 0% (0–98)	18/24, 75% (53–90)
Peritoneal dissemination	51/58, 88% (77–95)	2/8, 25% (3–65)	49/50, 98% (89–100)	2/3, 67% (9–99)	49/55, 89% (78–96)

Values in parentheses represent the 95% confidence interval

*PPV* positive predictive value, *NPV* negative predictive value, *NA* not available

**Table 3:** Diagnostic performance of imaging in preoperatively deemed locally early stage gastric-type adenocarcinoma and usual-type endocervical adenocarcinoma

	Accuracy	Sensitivity	Specificity	PPV	NPV
DLES-GEA (*n* = 36)					
Parametrial invasion	24/36, 67% (49–81)	2/14, 14% (2–43)	22/22, 100% (85–100)	2/2, 100% (16–100)	22/34, 65% (47–80)
Any vaginal invasion^a^	27/36, 75% (57–88)	4/13, 31% (9–61)	23/23, 100% (85–100)	4/4, 100% (40–100)	23/32, 72% (53–86)
Adnexal involvement	28/35, 80% (63–92)	0/7, 0% (0–41)	28/28, 100% (88–100)	0/0, NA	28/35, 80% (63–92)
Bladder invasion^a^	NA	NA	NA	NA	NA
Any rectal invasion^a^	35/36, 97% (85–100)	0/1, 0% (0–98)	35/35, 100% (90–100)	0/0, NA	35/36, 97% (85–100)
Lymph node metastasis					
Pelvic lymph node	27/35, 77% (60–90)	5/12, 42% (15–72)	22/23, 96% (78–100)	5/6, 83% (36–100)	22/29, 76% (56–90)
Paraaortic lymph node	10/13, 77% (46–95)	0/3, 0% (0–71)	10/10, 100% (69–100)	0/0, NA	10/13, 77% (46–95)
Peritoneal dissemination	34/36, 94% (81–99)	1/3, 33% (84–91)	33/33, 100% (89–100)	1/1, 100% (3–100)	33/35, 94% (81–99)
DLES-UEA (*n* = 136)					
Parametrial invasion	127/136, 93% (88–97)	1/8, 13% (0–53)	126/128, 98% (94–100)	1/3, 33% (5–83)	126/133, 95% (93–96)
Any vaginal invasion^b^	122/136, 90% (83–94)	4/12, 33% (10–65)	118/124, 95% (90–98)	4/10, 40% (18–67)	118/126, 94% (91–96)
Adnexal involvement	131/131, 100% (97–100)	1/1, 100% (3–100)	130/130, 100% (97–100)	1/1, 100% (3–100)	130/130, 100% (97–100)
Bladder invasion^b^	NA	NA	NA	NA	NA
Rectal invasion^b^	NA	NA	NA	NA	NA
Lymph node metastasis					
Pelvic lymph node	124/136, 91% (85–95)	8/17, 47% (23–72)	116/119, 97% (93–100)	8/11, 73% (44–90)	116/125, 93% (89–95)
Paraaortic lymph node^b^	NA	NA	NA	NA	NA
Peritoneal dissemination^b^	NA	NA	NA	NA	NA

Values in parentheses represent the 95% confidence interval

*PPV* positive predictive value, *NPV* negative predictive value, *DLES* deemed locally early stage, *GEA* gastric-type endocervical adenocarcinoma, *UEA* usual-type endocervical adenocarcinoma, *NA* not available

^a^There were no cases with lower one-third vaginal invasion, bladder invasion, or rectal mucosal invasion in the early stage GEA group

^b^There were no cases with lower one-third vaginal invasion, any bladder/rectal invasion, para-aorti lymph node metastasis, and peritoneal dissemination in the early stage UEA group

The accuracy and NPV of parametrial invasion, any vaginal invasion, and PELNM/PALNM were less than 0.80 both in the all-GEA group and the DLES-GEA group. Especially, the accuracy and NPV of parametrial invasion were less than 0.70 in both groups. Although the number was small, some factors were overestimated in certain cases. In the all-GEA group, the overestimations included parametrial invasion (*n* = 3), any vaginal invasion (*n* = 1), vaginal invasion of the lower third (*n* = 1), any bladder invasion (*n* = 2), PELNM (*n* = 2), PALNM (*n* = 1), and peritoneal dissemination (*n* = 1). In the DLES-GEA group, one case of PELNM was overestimated.

**Fig. 2:**
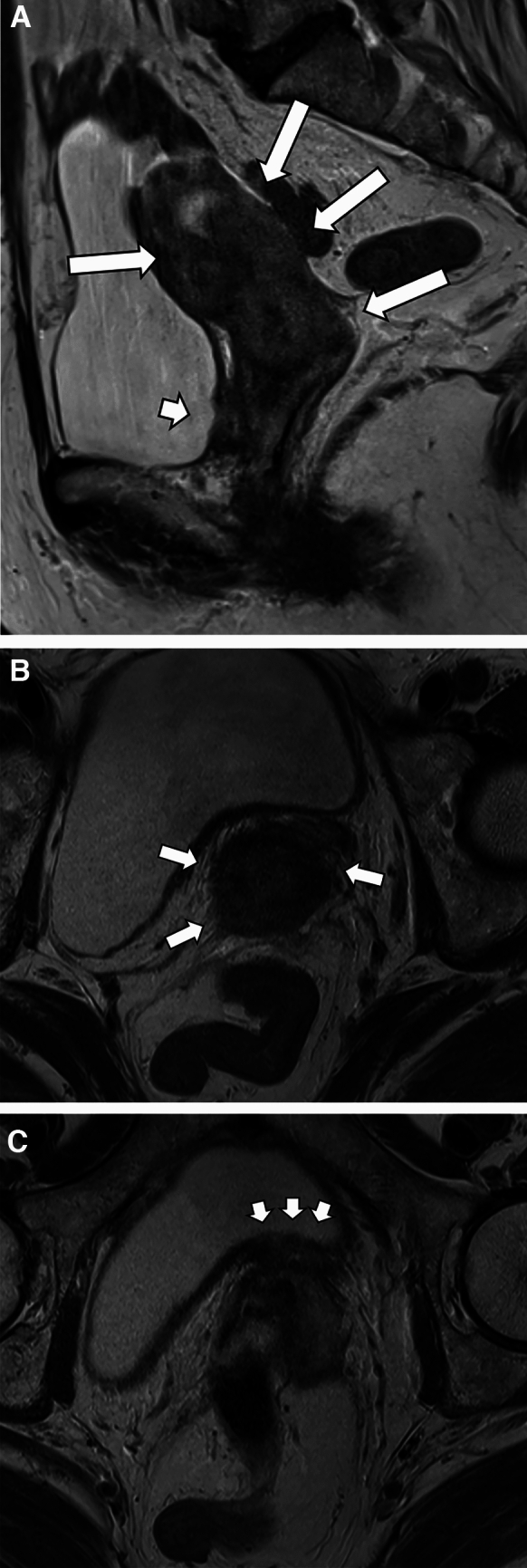
A representative case of unexpectedly advanced gastric-type endocervical adenocarcinoma in her 70s preoperatively diagnosed with FIGO IIB. MRI shows a cervical tumor extending to the lower uterine body (**A**, T2-weighted sagittal image, long arrows) and invading the anterior (**A**, a short arrow) and bilateral parametrium (**B**, T2-weighted oblique axial image: arrows). The tumor disrupts the bladder wall but does not extend into the lumen (**C**, T2-weighted oblique axial image, short arrows). In this case, no bladder mucosal invasion was diagnosed preoperatively, and cystoscopy also showed no evidence of mucosal invasion. Anterior pelvic exenteration was performed. It was pathologically staged as T4aN1: invasion to bilateral parametrium, mucosa of bladder and left ureter, and bilateral pelvic lymph node metastases

**Fig. 3:**
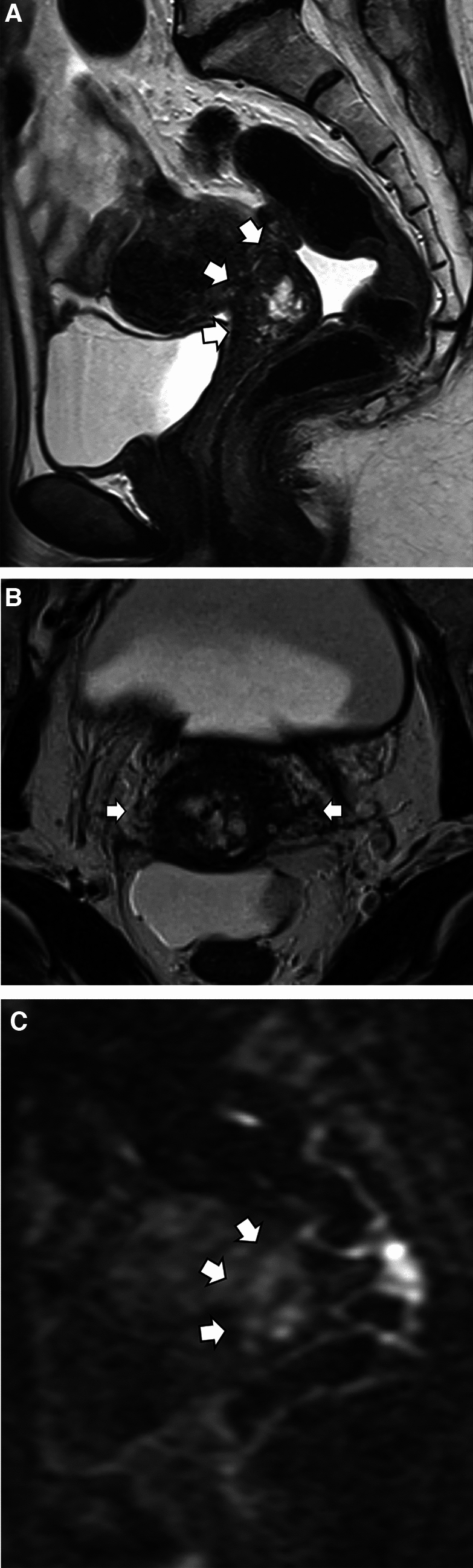
Representative case of unexpectedly advanced gastric-type endocervical adenocarcinoma in a woman in her 50s in the deemed locally early-stage group. MRI shows a tumor localized to the cervix (**A**, **B**, T2-weighted sagittal and oblique axial images, arrows). Diffusion-weighted images depict a mildly hyperintense tumor (**C**, arrows). In this case, preoperative clinical diagnosis was FIGO IB1 with no parametrial invasion. After radical hysterectomy, a pathological examination revealed T2bN1 (bilateral parametrial invasion and right pelvic lymph node metastasis) with peritoneal dissemination

**Fig. 4:**
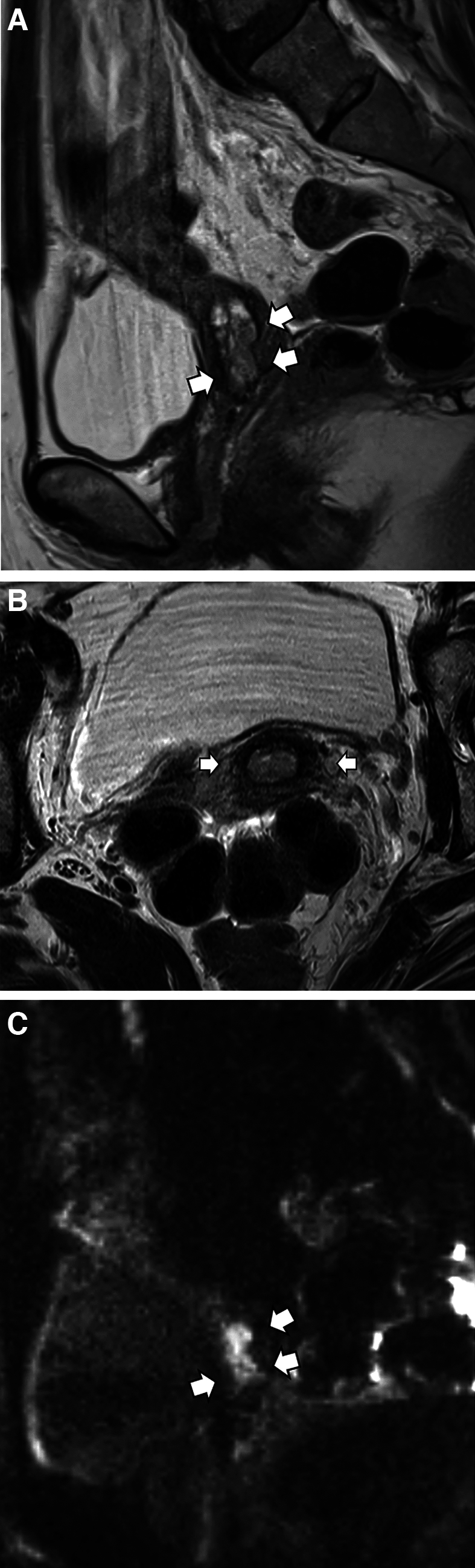
Representative case of usual-type endocervical adenocarcinoma in a woman in her 50s in the deemed locally early stage group. MRI shows a tumor localized to the cervix, without apparent parametrial invasion (**A**, **B**, T2-weighted sagittal and oblique axial images, arrows). Diffusion-weighted images clearly depict a hyperintense tumor (**C**, arrows). Preoperative clinical diagnosis was FIGO IB2, which was pathologically confirmed after radical hysterectomy

In the DLES-UEA group, the accuracy and NPV of all staging factors were greater than 0.90. The representative cases of advanced GEA, preoperatively deemed locally early stage but unexpectedly advanced GEA, and pathologically confirmed locally early stage UEA are presented in Figs. 2, 3, and 4.

## Comparison of preoperative imaging diagnostic performance for DLES-*GEA* and DLES-UEA

As shown in Table 3, the diagnostic performance for the DLES-GEA group was completely inferior to that for the DLES-UEA group. Regarding the proportion of underestimation, Fisher’s exact tests revealed significant differences in all staging factors but bladder/rectal invasion (very few or no patients in both groups) and PALNM (only 3 patients of the DLES-UEA group had undergone paraaortic lymphadenectomy) (Table 4). Regarding the underestimation of parametrial invasion, the power of the analysis for the difference in probability between the two groups was 0.98.

**Table 4:** Proportions of underestimation of preoperative imaging between deemed locally early stage gastric-type adenocarcinoma and usual-type endocervical adenocarcinoma

Factor	DLES-GEA group	DLES-UEA group	*p* value*
Parametrial invasion	12/34, 35%	7/133, 5%	< 0.001
Vaginal invasion	9/32, 28%	8/126, 6%	0.002
Adnexal involvement	7/35, 20%	0/130, 0%	< 0.001
Bladder invasion	0/36, 0%	0/136, 0%	NA
Rectal invasion	1/36, 3%	0/136, 0%	0.209
Lymph node metastasis			
Pelvic lymph node	7/29, 24%	9/125, 7%	0.014
Paraaortic lymph node	3/13, 23%	0/3, 0%	1.000
Peritoneal dissemination	2/35, 6%	0/136, 0%	0.041

*DLES* deemed locally early stage, *GEA* gastric-type endocervical adenocarcinoma, *UEA* usual-type endocervical adenocarcinoma, *NA* not available

*p* values were calculated by Fisher's exact test

## Discussion

This multi-center retrospective study revealed the limited diagnostic performance of preoperative imaging evaluations of tumor extension in GEA in an actual clinical setting. Especially, the sensitivity and NPV of respective FIGO staging factors were notably low. When compared with UEA, among patients with preoperatively diagnosed locally early stage disease, the proportions of underestimation of imaging for respective FIGO staging factors were significantly higher.

For evaluation of T factors in cervical cancer, MRI has been regarded as the key imaging modality with the highest precision. However, for GEA, the diagnostic performance of MRI, especially in terms of sensitivity and NPV, remains lower than that reported earlier, with no consideration of histological variation. Comparisons of sensitivity between our results in the all-GEA group and the meta-analysis were the following: parametrium, 0.49 (95% CI 0.31–0.66) vs. 0.71 (95% CI 0.62–0.79) [13] or 0.75 (95% CI 0.65–0.82) [14]; vagina, 0.54 (95% CI 0.34–0.72) vs. 0.71 (0.54–0.84) [13]; bladder mucosal invasion, 0.50 (95% CI 0.01–0.99) vs. 0.84 (0.57–0.95) [13]. There were no cases with rectal mucosal invasion in our cohorts. Comparison to the DLES-UEA group revealed significantly higher prevalence of underestimation of imaging in the DLES-GEA group. In clinical settings, difficulties in the pretreatment evaluation of T factors by imaging in GEA have been experienced, as well as by physical examinations. The endophytic and highly infiltrating growth pattern, rather than an exophytic or mass-forming pattern, is a distinct MRI feature that reflects its aggressive nature, which potentially leads to underestimation of the tumor extent [9–11, 15]. Significantly higher apparent diffusion coefficient values compared to squamous cell cervical cancer have been reported, suggesting a correlation with the histologically sparse distribution of tumor cells [15]. This might reduce the visibility of GEA on DWI, and affect the accuracy of tumor extent evaluation. Diagnostic criteria of T factors on MRI have been unavoidably developed corresponding to commonly observed histological types (squamous cell carcinoma and UEA), e.g., full-thickness disruption of the low T2-weighted signal intensity of cervical stroma for parametrial invasion [16]. Detecting subtle signal increases on T2-weighted images and/or DWI, as well as developing sequences that can sensitively detect these changes, might be useful for improving sensitivity and NPV. Further improvement of the diagnostic performance for GEA will necessitate the accumulation of clinical cases, with development and incorporation of new approaches particularly addressing GEA.

In addition to its local extension, our study revealed the low sensitivity of imaging for detection of lymph node metastasis (0.48 and 0.00 for PELNM and PALNM), adnexal involvement (0.15), and peritoneal dissemination (0.25). A meta-analysis reported the sensitivity of 0.65 (0.60–0.69) for FDG PET/CT and 0.54–0.63 for MRI in the diagnosis of lymph node metastasis [17]. It is noteworthy that the rate of ovarian metastasis in GEA patients (22% of All GEA group and 19% of the DLES-GEA group) was much higher than that of the DLES-UEA group (1%). That for overall adenocarcinoma has been reported as 6.3% [18]. Pretreatment imaging evaluation plays important roles in treatment decision making. The recognition of these pitfalls is expected to allow more sufficient preoperative preparation: multidisciplinary discussions, surgical planning considering multiple options, and flexible intraoperative approaches based on detailed preoperative explanations to patients. Our results are also expected to be important for the planning of chemoradiation therapy (CRT). Especially, the potential risk of underestimating PALNM, adnexal involvement and peritoneal dissemination should be examined for radiotherapy treatment planning.

Patients with preoperatively DLES-GEA had significantly higher proportions of underestimation of imaging, than those with DLES-UEA. An earlier multi-center study showed GEA as more significantly associated with pathological risk factors and poorer prognosis than UEA was [4]. In that study, they found no significant differences in prognostic outcomes between GEA and UEA among patients with pathological T1b > 4 cm (in AJCC TNM classification, T1b2 in ver. 8) and T2 [4]. Considering the higher risk of underestimation of imaging, as shown in our study, careful consideration is needed for interpreting their results in the preoperative prognostic prediction or in CRT when no pathological confirmation of the tumor extent is available. Further studies of GEA must be conducted, particularly addressing the association between imaging evaluation and prognosis.

This study has several limitations. First, this retrospective multi-center study was based on the medical charts and radiological reports in the actual clinical settings, instead of central imaging review. All MRI (and FDG PET-MRI), CT, and FDG PET-CT images were evaluated by board-certified radiologists. The quality was regarded as assured. We chose this approach to elucidate the current situation in practical clinical settings. Second, reevaluations by board-certified radiologists of respective centers specialized to gynecologic imaging were conducted, if not explicitly stated in the imaging reports or medical charts. This approach might differ slightly from actual clinical practice. However, considering the rarity of GEA and the limited number of patients, this method was selected to maximize the use of the available patient cohorts, rather than treating missing data as unavailable. Third, the cohorts of GEA and UEA constitute only patients treated by primary surgery. Therefore, there might be selection bias. Similarly, very low sensitivity and PPV for parametrial invasion in the DLES-GEA and DLES-UEA groups were regarded as biased by the inherent difficulty in detecting parametrial invasion within the populations including only those patients with preoperatively presumed cancer less than T2b.

In conclusion, the preoperative imaging diagnostic performance for staging factors in GEA did not meet clinical expectations, especially for sensitivity and NPV. Specifically, regarding patients with preoperatively DLES cancer, the proportions of underestimation in GEA were significantly higher than in UEA. Future incorporation of approaches specifically addressing GEA is desirable to improve diagnostic imaging performance.
